# HSulf-1 deficiency dictates a metabolic reprograming of glycolysis and TCA cycle in ovarian cancer

**DOI:** 10.18632/oncotarget.5605

**Published:** 2015-09-10

**Authors:** Susmita Mondal, Debarshi Roy, Juliana Camacho-Pereira, Ashwani Khurana, Eduardo Chini, Lifeng Yang, Joelle Baddour, Katherine Stilles, Seth Padmabandu, Sam Leung, Steve Kalloger, Blake Gilks, Val Lowe, Thomas Dierks, Edward Hammond, Keith Dredge, Deepak Nagrath, Viji Shridhar

**Affiliations:** ^1^ Department of Experimental Pathology, Mayo Clinic College of Medicine, Rochester, MN, USA; ^2^ Department of Anesthesiology, Mayo Clinic College of Medicine, Rochester, MN, USA; ^3^ Department of Chemical and Biomolecular Engineering, Rice University, Houston, TX, USA; ^4^ Department of Pathology and Laboratory Medicine, University of British Columbia, Canada; ^5^ Department of Nuclear Medicine, Mayo Clinic College of Medicine, Rochester, MN, USA; ^6^ Department of Chemistry, Biochemistry I, Bielefeld University, Bielefeld, Germany; ^7^ Institute of Medical Biochemistry Leopoldo de Meis, Federal University of Rio de Janeiro, Rio de Janeiro, RJ, Brazil; ^8^ Progen Pharmaceuticals Ltd, Brisbane, Queensland, Australia

**Keywords:** HSulf-1, Warburg effect, HB-EGF, ovarian cancer, c-Myc, PG545

## Abstract

Warburg effect has emerged as a potential hallmark of many cancers. However, the molecular mechanisms that led to this metabolic state of aerobic glycolysis, particularly in ovarian cancer (OVCA) have not been completely elucidated. HSulf-1 predominantly functions by limiting the bioavailability of heparan binding growth factors and hence their downstream signaling. Here we report that HSulf-1, a known putative tumor suppressor, is a negative regulator of glycolysis. Silencing of HSulf-1 expression in OV202 cell line increased glucose uptake and lactate production by upregulating glycolytic genes such as Glut1, HKII, LDHA, as well as metabolites. Conversely, HSulf-1 overexpression in TOV21G cells resulted in the down regulation of glycolytic enzymes and reduced glycolytic phenotype, supporting the role of HSulf-1 loss in enhanced aerobic glycolysis. HSulf-1 deficiency mediated glycolytic enhancement also resulted in increased inhibitory phosphorylation of pyruvate dehydrogenase (PDH) thus blocking the entry of glucose flux into TCA cycle. Consistent with this, metabolomic and isotope tracer analysis showed reduced glucose flux into TCA cycle. Moreover, HSulf-1 loss is associated with lower oxygen consumption rate (OCR) and impaired mitochondrial function. Mechanistically, lack of HSulf-1 promotes c-Myc induction through HB-EGF-mediated p-ERK activation. Pharmacological inhibition of c-Myc reduced HB-EGF induced glycolytic enzymes implicating a major role of c-Myc in loss of HSulf-1 mediated altered glycolytic pathway in OVCA. Similarly, PG545 treatment, an agent that binds to heparan binding growth factors and sequesters growth factors away from their ligand also blocked HB-EGF signaling and reduced glucose uptake *in vivo* in HSulf-1 deficient cells.

## INTRODUCTION

Cancer cell survival and growth vastly depends on the deregulated overlapping signaling cascades which are initiated at the extracellular matrix by a range of growth factors [1]. Constitutive activation of these signals instructs the cells for continual biomass production to accumulate metabolic intermediates as the source of building blocks. Mounting evidence indicates that this altered cell metabolism can be actively regulated by oncogenes and tumor suppressors. Among the tumor suppressors, the roles of P53 and PTEN leading to metabolic reprogramming of cancer are well documented [2, 3]. However, it is unclear whether other putative tumor suppressors also play a critical role in altering the Warburg effect. Thus, a comprehensive understanding of the molecular alterations by other putative tumor suppressors or oncogenes that lead to altered metabolism may not only shed new light on tumorigenesis but also provide novel strategies for therapeutically targeting these alterations.

Majority of the growth factors like FGF-1,-2 [4], VEGF [5], HB-EGF [6] and cytokines including IL-6 [7] and IL-8 [8] depend on heparan sulfate proteoglycans (HSPGs) for binding and signaling through their cognate receptor. Heparan sulfate (HS) is a glycosaminoglycan consisting of repeating unbranched negatively charged disaccharide units variably sulfated at the 3-*O*, 6-*O*, or N sites on glucosamine and the 6-*O* site on glucuronic/iduronic acid [9]. Growth factors and cytokines form the ternary complexes with their cognate receptors and HS resulting in ligand-mediated activation. We had previously reported that HSulf-1 (also known as Sulfatase 1, Sulf-1, KIAA1077 and Extracellular Sulfatase Sulf-1 [6] is downregulated in a majority of ovarian cancer cell lines [6] (Figure S1) and tumors including serous, endometrioid and clear cell tumors of the ovary [10]. Also, we have demonstrated that loss of HSulf-1modulates the signaling of HS binding growth factors such as FGF-2, VEGF, HGF, and HB-EGF in ovarian [11], head and neck squamous carcinoma [11] and metastatic breast carcinomas [12] respectively and plays an important role in tumor progression, metastasis and angiogenesis [10, 13, 14]. Further, we showed that HIF-1α, a major transcription factor that also promotes altered metabolic signature, negatively regulates HSulf-1 expression in breast cancer [15]. Moreover, HSulf-1 silencing increases the ability to form anchorage-independent colonies *in vitro* and enhanced tumorigenicity *in vivo* [16]. Other studies also demonstrated that HSulf-1 blocks cell proliferation, migration and growth *in vitro* and *in vivo* in hepatocellular carcinoma [17, 18] and suppresses the malignant growth in lung and gastric cancer by inhibiting ERK, AKT and hedgehog signaling respectively [19, 20]. Based on these findings, we hypothesized that HSulf-1, due to its regulation of growth factor mediated signaling, might play a critical role in altering cellular metabolism. Indeed, our recent findings demonstrate that loss of HSulf-1 potentially contributes to the metabolic alterations in the lipogenic phenotype of ovarian cancer cells [21]. Here, we investigated whether HSulf-1 deficiency would also affect other metabolic pathways such as glycolysis and TCA cycle. By combining bioenergetics and metabolic studies, our results show for the first time that growth factor signaling modulated by HSulf-1 loss increases glucose uptake and lactate production and alters the energy metabolism and subsequently promoting c-Myc activation. In this study we utilized PG545, a novel synthetic agent currently in Phase 1 clinical trials (Clinical Trials gov.identifier, NCT02042781) and essentially mimics HSulf-1 function. PG545 functions as HS mimetic by simultaneously blocking HS-mediated growth factor signaling leading to inhibition of angiogenesis and carcinogenesis [22-24] including in ovarian cancer [22]. However, whether PG545 also modulates the glycolytic phenotype has never been explored before. We show for the first time that PG545 treatment resulted in significant reduction in glycolytic phenotype induced by loss of HSulf-1 and downregulated c-Myc and expression of key glycolytic enzymes *in vitro*. More importantly, PG545 decreased *in vivo* glucose uptake, lactate production and markedly inhibited tumor progression.

## RESULTS

### HSulf-1 reprograms the glycolytic pathway

**Figure 1:**
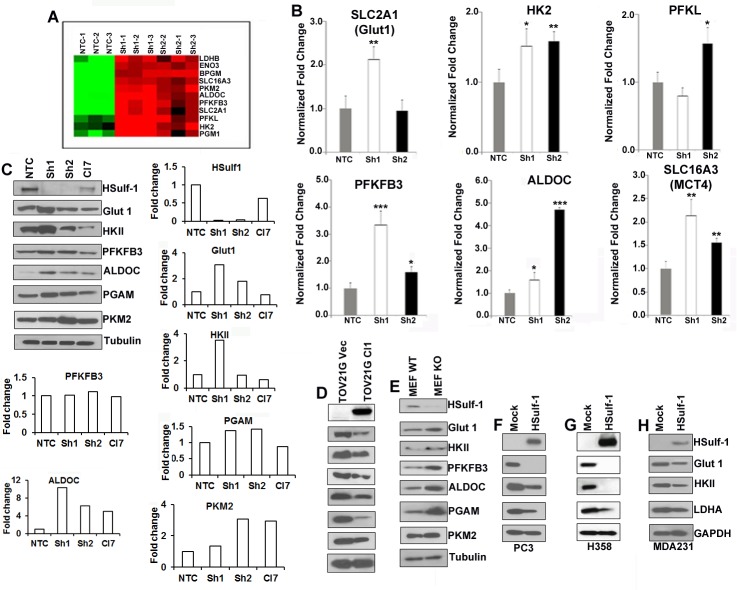
Absence of HSulf-1 augmented glycolytic key enzymes **A.** Microarray analysis of glycolytic genes in NTC, Sh1and Sh2 cells in triplicates. **B.** QRT-PCR analysis of relative mRNA levels. **C.** Immunoblot analysis of glycolytic enzymes in NTC, Sh1, Sh2 and Cl7 cells (*N* = 2). Fold change as determined by densitometric analysis of these enzymes in Sh1 and 2 cells compared to NTC cells are shown as bar graph. Immunoblot analysis of glycolytic enzymes in TOV21GVec and Clone 1 cells **D.**, in WT and HSulf-1 KO MEF cells **E.**. Protein levels of glycolytic enzymes after transient over-expression of HSulf-1 in PC3 **F.**, H358 **G.** and MDA231 **H.** cells.

To understand the impact of HSulf-1 on glycolytic metabolism in OVCA cells, we analyzed the levels of glycolytic genes of OV202 non-targeted control (NTC) and HSulf-1-ShRNA downregulated clones, Sh1 and Sh2 cells [16]. The microarray analysis (accession no- GSE67086) revealed aberrant glycolytic gene expression in Sh1 and Sh2 compared to NTC cells (Figure 1A). Q-PCR validation showed that glycolytic genes including hexokinase II (HKII), 6-phosphofructo-2-kinase/fructose-2,6-biphosphatase 3 (PFKFB3), 6-phosphofructokinase, liver type (PFKL), aldolase C, fructose-bisphosphate (ALDOC) and related genes including glucose transporter 1 (Glut1), and moncarboxylase transporter 4 (MCT4) were upregulated in Sh1 and Sh2 cells (Figure 1B). Similar results were also observed at the protein level by immunoblot including PGAM and PKM2. Importantly, most of the protein levels were rescued after re-expression of HSulf-1 in Sh1 (Cl7) cells (Figure 1C).

To determine if ectopic expression of HSulf-1 will reduce glycolytic enzyme levels, we generated a HSulf-1-overexpressing stable clone in TOV21G (Figure 1D). Results showed that enhanced HSulf-1 expression in TOV21G clonal line (Cl1) resulted in the downregulation of all of these enzymes compared to vector control (Figure 1D). To further substantiate loss of HSulf-1 induced alterations in glycolysis, we checked the enzyme levels in immortalized Wild-Type (WT) and HSulf-1 Knock-Out (KO) MEFs and observed higher Glut 1, HKII and LDHA levels in MEF KO cells (Figure 1E).

To assess the role of putative tumor suppressor HSulf-1 in other cancer, we transiently overexpressed HSulf-1in prostate cancer (PC3), lung cancer (H358) and breast cancer (MDA-231) cells. Results showed a marked reduction in Glut1, HKII and LDHA (Figures 1F-1H) in HSulf-1 expressing cells compared to vector transfected controls. These results suggested that HSulf-1 might play a significant role in promoting glycolysis in cancer.

### Loss of HSulf-1 induced enhanced glycolytic phenotype in ovarian cancer *in vitro* and *in vivo*

**Figure 2:**
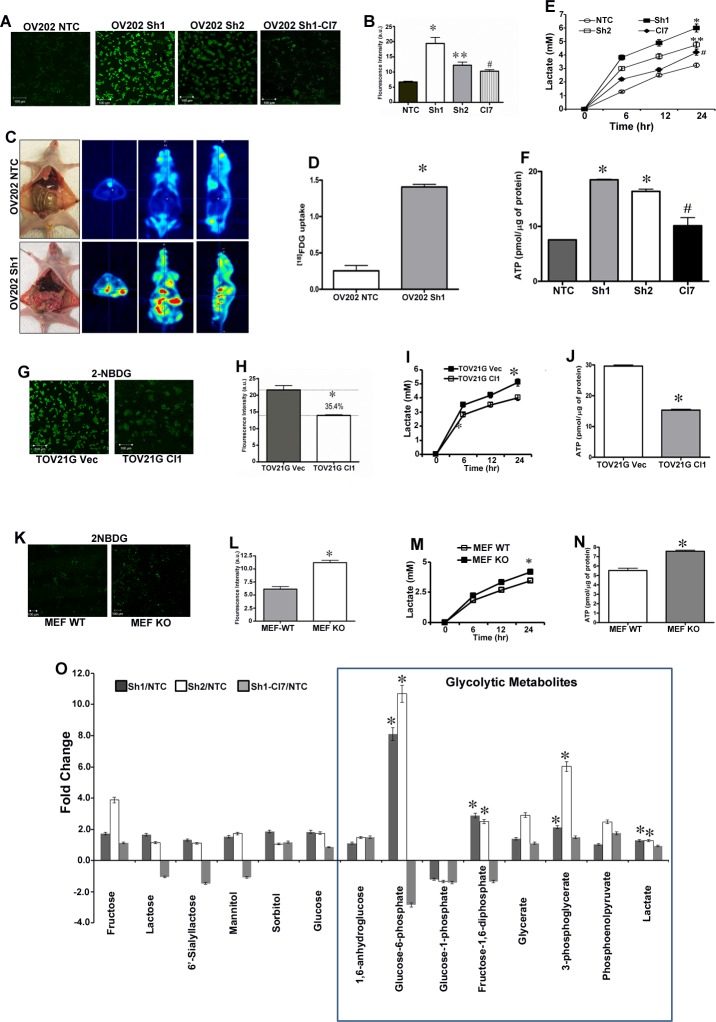
HSulf-1 loss induces enhanced glycolytic phenotype in ovarian cancer **A.** Live cell imaging showing glucose uptake in NTC, Sh1, Sh2 and Cl7 using 2-NBDG. **B.** Quantification of fluorescence intensity of the glucose uptake in OV202NTC, Sh1, Sh2 and Cl7 cells. **C.** *In vivo* glucose uptake using ^18^FDG in OV202NTC and Sh1 xenografts. **D.** *In vivo* glucose uptake was calculated using PMOD Biomedical Image Quantification software, *p ≤ 0.001. **E.** Lactate secretion in media of OV202NTC, Sh1, Sh2 and Cl7 cells, **F.** Intracellular ATP levels in OV202NTC, Sh1, Sh2 and Cl7 cells, (*p and **p < 0.05 compared to NTC, #*p* < 0.05 compared to Sh1). Glucose uptake **G.**,glucose uptake intensity **H.**, lactate secretion **I.**,and intracellular ATP levels **J.** in TOV21G Vec and Cl cells where *p < 0.05. Glucose uptake **K.**, its intensity **L.**, lactate secretion **M.**, and intracellular ATP levels **N.** in WT and KO MEFs, where *p < 0.05. **O.** Fold increase/decrease of glycolytic metabolite were evaluated using GC/MS and LC/MS/MS platforms and fold change were calculated by the average metabolite level ofSh1/NTC, Sh2/NTC and Sh1-Cl7/Sh1. *p <0.05.

To evaluate if upregulation of glycolytic genes and enzyme levels upon HSulf-1 loss resulted in increased glucose uptake, we stained the live cells using 2-NBDG a fluorescent glucose analogue which is used to monitor glucose uptake in live cells. Data shows that glucose uptake was enhanced in Sh1 and Sh2 cells by 2.8 and 1.8 fold respectively as compared to NTC cells (Figures 2A-2B). Interestingly, re-expression of HSulf-1 resulted in significant reduction in glucose uptake in Cl7compared to Sh1 cells (Figures 2A-2B). Moreover, *in vivo* imaging using^18^FDG-PET showed a marked increase in glucose uptake in 2 week old Sh1-xenograft but not in the NTC-xenograft model (Figures 2C-2D, S2). Our results also demonstrated a significant increase in lactate secretion and higher levels of ATP in Sh1 and Sh2 compared to NTC cells, while this phenotype is reversed in Sh1-Cl7 (Figures 2E-2F). Conversely, forced expression of HSulf-1 in TOV21G (Cl1) showed a significant reduction (35.4%) in glucose uptake, lactate production (21.5%) and ATP levels (50%) (Figures 2G-2J). Similarly, HSulf-1 KO MEFs showed 84% increase in glucose uptake when compared to WT-MEFs (Figures 2M-2N), and exhibited 22% increase in lactate secretion and 37% increase in ATP content (Figures 2K-2N), further substantiating that loss of HSulf-1-induced aerobic glycolysis. Also, transient overexpression of HSulf-1 in PC3, H358 and MDA231 cancer cell lines reduced lactate secretion (Figure S3). Additionally, our metabolite analysis demonstrated that HSulf-1 deficiency was closely associated with increased levels of glycolytic metabolites including glucose 6-phosphate [fold change (FC) compared to NTC (FC =10.68 *p* =< 0.001)], fructose 1,6 bisphosphate (FC=2.89, p=0.03), 3-phosphoglycerate (FC = 6.02, *p* = 0.01) and intracellular lactate (FC = 1.5, *p* = 0.002) (Figure 2O). Altogether, these results suggest a key role of HSulf-1 in modulating the glycolytic phenotype.

### HSulf-1 decouples the energy flow from glycolysis to TCA cycle

**Figure 3:**
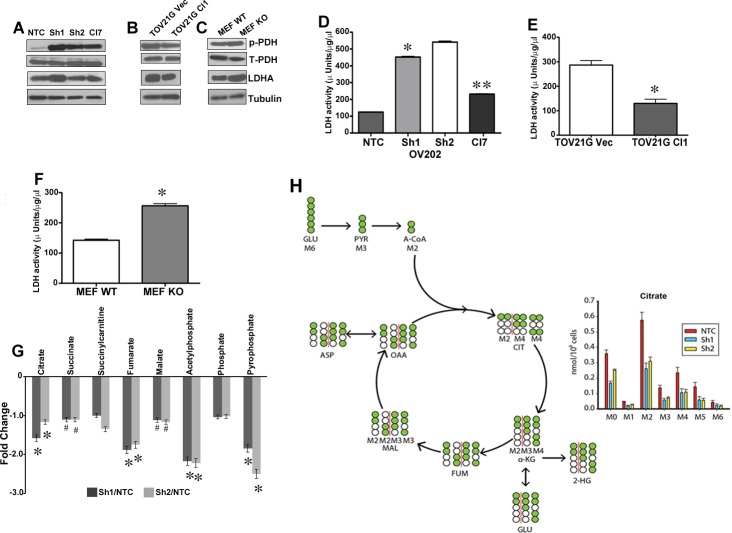
Disruption of energy flow from glycolysis to TCA cycles Immunoblot analysis of p-PDH, PDH, and LDHA in OV202 clonal cells **A.**, TOV21G vec and Cl1 **B.** and HSulf-1 WT and KO MEF cells **C.**. LDH activity in OV202 clonal lines **D.**, TOV21G vec and Cl1 **E.** and in MEFs **F.** where*p< 0.05 and **p< 0.05 compared to Sh1 cells and /or compared to vector transfected and WT-MEF cells respectively. **G.** Fold increase/decrease of TCA metabolites evaluated on metabolon platform and fold change were calculated by the average metabolite level of Sh1/NTC, Sh2/NTC and Sh1-Cl7/Sh1, *p < 0.05 and #*p* < 0.9. **H.** Isotope tracer analysis using ^13^C_6_ labeled glucose to estimate glucose's contribution towards TCA cycle metabolites level. Data is normalized by cell number for OV202 NTC, Sh1 and Sh2 cells (*n* = 7).

To elucidate the role of HSulf-1 in the next level of glucose oxidation, we examined the inhibitory phosphorylation of PDH (S-293) along with PDK1 expression as PDH and PDK regulates the entry of acetyl CoA into TCA cycle [23]. Our results showed increased p-PDH in E1 complex in Sh1 and Sh2 compared to NTC cells (Figure 3A). Similar results were observed in TOV21G Vec/Cl1 (Figure 3B) and in MEFs (Figure 3C). It is well established that increased levels of pyruvate that accumulate due to higher aerobic glycolysis is either converted to acetyl CoA or lactate [24]. Since the level of p-PDH were increased with loss of HSulf-1, we further monitored the activities and levels of LDH, a key enzyme involved in conversion of pyruvate to lactate in order to maintain high glycolytic rate. HSulf-1 downregulated Sh1 and Sh2 clones showed a marked increase in LDHA expression (Figure 3A) and activity (Figure 3D). Moreover, we found that with the HSulf-1 re-expression, LDHA expression and activity were reduced in Cl7 cells (Figure 3A and 3D). Likewise, we found reduced LDHA level (Figure 3B) and activity (Figure 3E) in the HSulf-1 over expressing TOV21GCl compared to vector. Similar results were also observed in HSulf-1 KO MEF compared to MEF WT cells (Figure 3C and 3F).

We next investigated whether HSulf-1 deficiency reduced glucose's contribution into TCA cycle. To this end, we performed unbiased metabolic profiling of TCA metabolites using the Metabolon platform (Metabolon Inc, NC) in NTC, Sh1, Sh2 and Cl7 cells. Results showed a significant reduction of most of the TCA metabolites including citrate, fumarate, malate and succinate in cells with loss of HSulf-1 (Figure 3G). To further demonstrate that loss of HSulf-1 reduces TCA cycle fluxes in ovarian cancer cells, we performed 13-C GC-MS based isotope tracer analysis using labeled U-^13^C_6_ glucose to reveal the distribution of metabolites in this metabolic pathway. As seen in Figure 3H, M6 (6 carbon labeled) glucose is converted into M3 pyruvate and then M2 acetyl-CoA through pyruvate dehydrogenase. M2 acetyl-CoA, combining with unlabeled oxaloacetate (M0 OAA) derived from other nutrient sources, is then condensed into M2 citrate. Citrate is decarboxylated into α-ketoglutarate, and then into additional TCA cycle metabolites. The abundance of each isotopomer metabolite was used to illustrate the glucose contribution to TCA cycle fluxes in ovarian cancer cells. Figure 3H showed that complete loss of HSulf-1 resulted in decreased glucose contribution to the majority of TCA cycle metabolites (citrate, fumarate, malate,aspartate) (Figure S4). The M2 citrate level, directly reflecting glucose's entry flux into TCA cycle, decreased from 0.58nmole per million of control cells to around 0.3nmole per million for both Sh1 and Sh2 cells (Figure 3H). Similar trends were also observed with other metabolites including fumarate, malate, and aspartate. However, the ^13^C-labeled metabolites levels of α-ketoglutarate, 2-hydroxyglutarate, and glutamate were unchanged. Collectively, these results suggest that HSulf-1 loss reduced the glucose contribution to TCA in mitochondria.

### Loss of HSulf-1 is associated with reduced oxidative phosphorylation (OXPHOS) and impaired mitochondria

**Figure 4:**
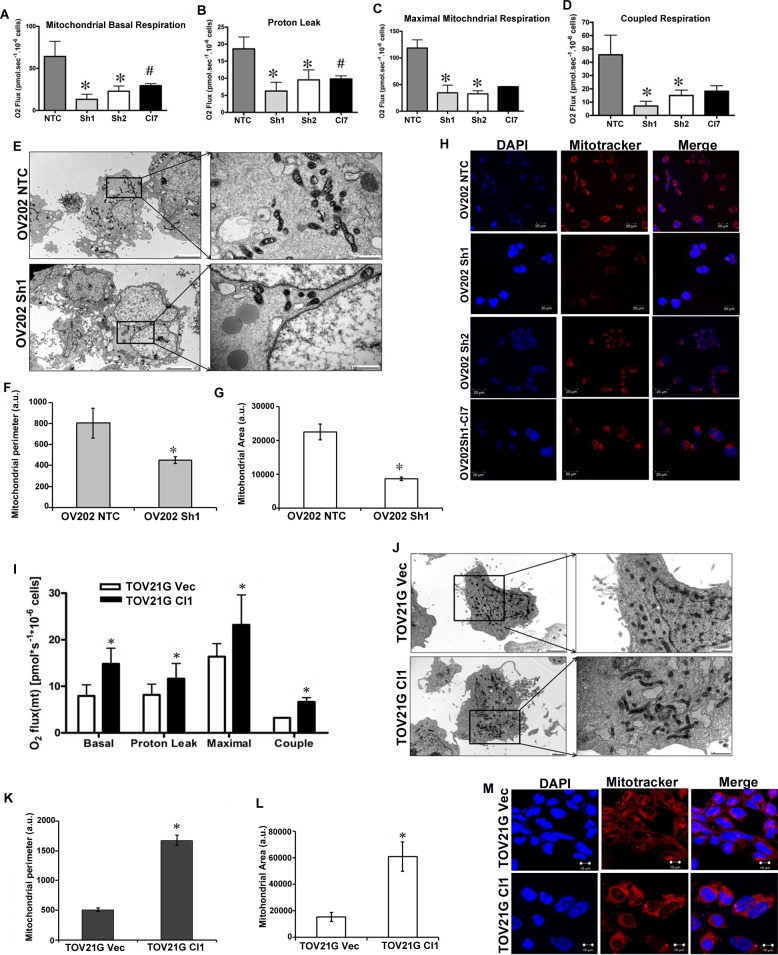
HSulf-1 is associated with reduced OXPHOS and impaired mitochondria **A.** Basal oxygen consumption in OV202 clonal cells. Oligomycin, FCCP and Rotenone were added subsequently to measure leak **B.**, maximal **C.** and coupled respirations **D. E.** Cultured NTC and Sh1cells were photographed following TEM at 5000X and in inset 25000X magnification to show altered mitochondrial morphology. Mitochondrial perimeter **F.** and area **G.** were calculated using Image J software from TEM micrographs in 10 cells from three different micrographs, *p < 0.05. **H.** Mitotracker staining of OV202 clonal cells. **I.** Basal, leak, maximal and coupled respiration rates in TOV21G Vec and Cl1 cells. **J.** Electron micrographs of TOV21G Vec and Cl1 cells with 5000X and in inset 25000X magnification. Mitochondrial perimeter **K.** and area **L.** were calculated in TOV21G Vec and Cl using Image J software from TEM micrographs in 10 cells from three different micrographs, *p < 0.05. **M.** Mitotracker staining of TOV21G Vec and Cl cells.

In order to investigate if the increased glycolytic flux and altered state of TCA cycle caused by the loss of HSulf-1 would have any impact on mitochondrial function, we measured the oxygen consumption rate (OCR). High resolution respirometry measurements in intact cells showed lower basal OCR in cells lacking HSulf-1 (Figure 4A). Sh1 and Sh2 cells showed reduction in OCR in all respiratory states compared to the controls (Figures 4B-4D). Moreover, these cells showed a lower coupled respiration rate compared to cells expressing HSulf-1, suggesting a regulatory role of HSulf-1 in mitochondrial ATP production (Figure 4D). Re-expression of HSulf-1 rescues the OCR to a level closer to NTC cells, suggesting that HSulf-1 might regulate mitochondrial oxidative phosphorylation. Moreover, Transmission Electron Microscopy (TEM) images clearly showed morphological alterations (Figure 4E) in mitochondria, reduced mitochondrial perimeter (Figure 4F), reduced area (Figure 4G) and less functional mitochondria as evaluated by mitotracker staining with loss of HSulf-1 in Sh1 (Figure 4H).

Conversely, enhanced expression of HSulf-1 in TOV21G Cll clearly showed higher OCR in all respirometry states compared to its vector (Figure 4I). The TOV21G Cll cells also recovered the morphological features (Figures 4J-4L) and functionality of mitochondria (as evidenced by mitotracker staining in TOV21G vector transfected compared to HSulf-1 transfected cells (Figure 4M). Consistent with these findings, HSulf-1 KO MEFs had lower OCR compared to WT MEF cells (Figure S5), suggesting a direct relationship between HSulf-1 loss and lower OXPHOS and mitochondrial alterations.

### HSulf-1 deficiency altered glucose metabolism through c-Myc induction

**Figure 5:**
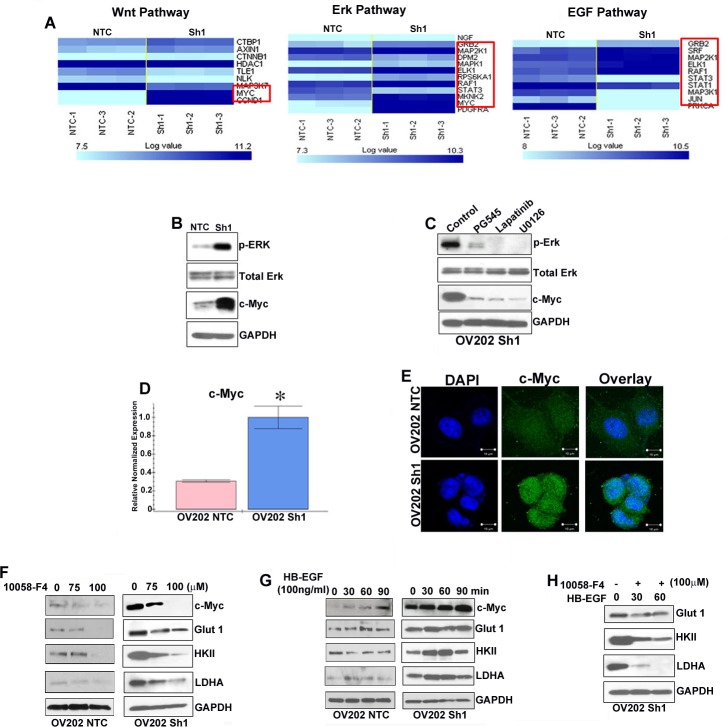
c-Myc is important for driving glycolysis in HSulf-1-deficient cells **A.** Normalized unsupervised hierarchical clustering of Wnt, ERK and EGF pathway genes for NTC and Sh1, each class representing three biological replicates. **B.** Immunoblot analysis of p-ERK, ERK and c-Myc in NTC and Sh1. **C.** Cell lysate from 24 hr treatment with PG545 (20μM), Lapatinib (20nM) and U1026 (20μM) were immunoblotted against p-ERK, ERK and c-Myc. **D.** QRT-PCR analysis of c-Myc in NTC and Sh1 cells, *P ≤ 0.001. **E.** NTC and Sh1 cells were immuno-stained with anti-c-Myc antibody (green) and mounted with DAPI and photographed using Zeiss LSM 510 confocal microscope. OV202NTC and Sh1 cells were treated with increasing concentration of c-Myc inhibitor for 24 hr and **F.** with HB-EGF for indicated time and immunoblotted **G.** for c-Myc, Glut 1, HKII and LDHA (*N* = 2). H. Sh1 cells were serum starved for 2 hr, treated with c-Myc inhibitor (10058-F4) for another 2 hr and then treated with HB-EGF for indicated time points. Protein lysates from each condition were probed against Glut 1, HKII and LDHA, where GAPDH served as loading control.

Our gene microarray and ingenuity pathway analysis in NTC and Sh1 cells [6] demonstrated that the genes in Ras-Raf-MAPK/ERK pathway were expressed at a much higher levels in the Sh1 and-2 cells, suggesting a major involvement of Ras-Raf-MAPK/ERK pathway in the survival/growth of cells in the absence of HSulf-1 (Figure 5A). Validation of microarray results with immunoblot analysis revealed a robust increase in ERK phosphorylation and c-Myc expression in Sh1 cells (Figure 5B). More importantly, HS-mimetic PG545, EGFR inhibitor lapatinib and MEK inhibitor U0126 attenuated ERK phosphorylation and c-Myc expression (Figure 5C), indicating an involvement of Raf-MAPK/ERK mediated c-Myc activation. Furthermore, c-Myc mRNA expression was 2.5 fold higher with enhanced cytoplasmic and nuclear localization of c-Myc in Sh1 cells compared to the NTC cells respectively (Figures 5D-5E). To determine if c-Myc could potentially play a role in this metabolic alteration, we used a specific c-Myc inhibitor (10058-F) and observed a dose dependent decrease in Glut 1, HKII and LDHA expression in Sh1 cells (Figure 5F, panel 2). However, Glut1 and HKII levels were downregulated only at the highest concentration of the c-Myc inhibitor (100μM) in the NTC cells with minimal changes in LDHA levels. To correlate whether HB-EGF mediated enhanced growth factor signaling in Sh1 cells results in higher c-Myc levels, we exogenously added HB-EGF and showed a time dependent increase of c-Myc expression along with augmented Glut 1, HKII and LDHA expression (Figure 5G). Pre-treatment of 10058-F reduced the HB-EGF mediated increment of Glut 1, HKII and LDHA levels (Figure 5H). These data suggests that loss of HSulf-1 can has a more pronounced effect in activating c-Myc through HB-EGF mediated ERK activation and subsequently reprograms the energy metabolism.

### Tumor development and metabolic alteration by loss of HSulf-1 is attenuated by HS mimetic PG545

**Figure 6:**
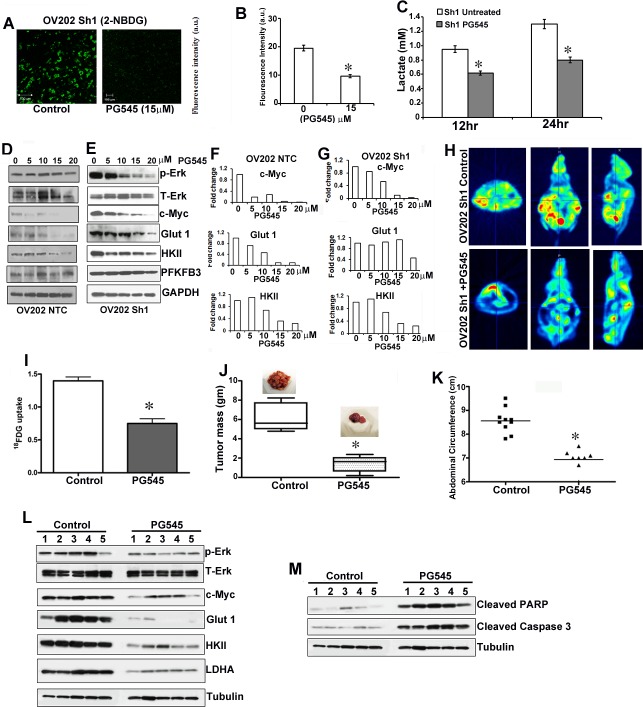
PG545 diminished HSulf-1-deficiency induced glycolysis and tumor growth **A.** Glucose uptake in live Sh1cells after PG545 (15 μM) treatment for 1 hr. **B.** Fluorescent intensity of glucose uptake in control and PG545 treated Sh1 cells. **C.** Lactate secretion after 12 and 24 hr PG545 treatment in Sh1 cells. **D.** and **E.** Immunoblot analysis of OV202 NTC and Sh1 cells treated with PG545 (0-20μM) for 24 hr. **F.** and **G.** Augmented downregulation of c-Myc, Glut1 and HKII in OV202 NTC cells compared to Sh1 cells following PG545 treatment shown as fold change respectively. **H.** Micro-PET imaging of ^18^FDG uptake in control and PG545 (20mg/kg, i.p.) treated mice. A representative image is provided in the transverse, coronal, and sagittal regions of interest in the tumor. I.^18^FDG uptake intensities were calculated in at least 20 planes and analyzed using PMOD Software, *P ≤ 0.01. **G.** A separate experiment with randomized tumor-bearing mice (*n* = 10), were treated with water or PG545 (20mg/kg) twice a week and after 28 days mice were euthanized and imaged. **J.** Excised Tumor weight from control and PG545 treated mice, *P ≤ 0.001. **K.** Abdominal circumference from untreated and PG545 treated mice (*n* = 10) measured at the day of sacrifice.*P ≤ 0.001. **L.**-**M.** Tumor lysates from control (*n* = 5) and PG545 treatment (*n* = 5) were selected randomly and immunoblotted.

To better understand the role of the putative tumor suppressor HSulf-1 in HS-dependent growth factor signaling, and the subsequent metabolic reprogramming and pro-tumorigenicity, we treated Sh1 cells with PG545, which mimics the action of HSulf-1. PG545 treatment significantly reduced glucose uptake (Figures 6A-6B) and lactate production (Figure 6C) in these cells. Moreover, PG545 dose-dependently reduced the phosphorylation of ERK and the levels of Glut1, HKII, and LDHA more in NTC cells expressing HSulf-1 compared to Sh1 cells (Figures 6D and 6E respectively). Densitometric analysis of the fold change in the levels of cMyc, Glut1 and HKII in NTC and Sh1 cells is shown as bar graph following PG545 treatment in Figures 6F and 6G. We postulated that PG545 might attenuate glucose uptake in *vivo*, as PG545 treatment decreased c-Myc activation through ERK inhibition reduced glucose uptake and Glut 1 expression *in vitro*. ^18^FDG-PET imaging in control and PG545 treated Sh1-xenografts showed a 50% reduction in ^18^FDG uptake in PG545 treated mice (Figures 6H and 6I). These findings demonstrated that PG545 selectively suppresses glucose uptake *in vivo*.

In a parallel experiment, PG545 treatment markedly decreased the size and number of metastatic nodules compared to control mice (Shown as inset in Figure 6J). The mean excised tumor weight was reduced by 65.04% in PG545 treated mice compared to control (Figure 6J). Moreover, the abdominal circumference which reflects the tumor burden of the peritoneum was significantly less in the treated mice compared with untreated mice (Figure 6K). To test whether PG545 abrogated the HB-EGF mediated signaling and subsequently enhanced glycolysis, we determined the expression of the glycolytic enzymes, p-ERK and cMyc in the xenografts of control untreated and PG545 treated mice. Western blot analysis showed that PG545 significantly reduced the expression of p-ERK, c-Myc and the levels of Glut 1, HKII and LDHA in the tumor lysates (Figure 6L). Additionally, increase in cleaved PARP and cleaved capase3 in PG545 xenografts indicate that the reduction in the tumor growth could be the result of increased apoptosis in these cells (Figure 6M).

## DISCUSSION

Increasing evidence suggest that the Warburg effect [25, 26] is common in cancer and is driven by oncogene addiction/tumor suppressor loss or mutations in the mitochondrial enzymes [27]. In this study, we report that HSulf-1 loss promotes glycolysis and impairs mitochondrial function leading to a significant reduction in OXPHOS. This phenotype is coupled to increased growth factor mediated ERK activation resulting in the upregulation of c-Myc activation. The frequent loss of HSulf-1 in OVCA may thus represent a genetic alteration promoting metabolic phenotype of OVCA.

Using HSulf-1 ShRNA downregulated OV202 cells and a clonal line of TOV21G with enhanced HSulf-1expression, we clearly showed modulation of the glycolytic pathway both *in vitro* and *in vivo*. Additionally, transient overexpression of HSulf-1 in prostate, lung and breast cancer cell lines reverses the glycolytic phenotype, suggesting a critical role of HSulf-1 in metabolic rearrangement in cancer. Glycolysis and mitochondrial respiration are tightly coupled processes. A key branch point in the glycolytic pathway is the production of pyruvate, which under anaerobic conditions is metabolized to lactate by LDH [28] and in normoxia by PDH to form acetyl-CoA [23]. The conversion of pyruvate to acetyl CoA by PDH is a crucial event which directs the energy flow from glycolysis to the TCA cycle [24]. Phosphorylation in the E1 complex of PDH by PDK inhibits its activity and energy flow to the mitochondria. In most cancers, LDHA is highly expressed and diverts the energy flow from mitochondria to from lactate. Indeed, our results demonstrated that silencing of HSulf-1 markedly enhanced LDHA level and activity in Sh1 and HSulf-1 KO MEF cells, whereas re-expression of HSulf-1 in Sh1 cells Cl7 resulted in the reduction of LDHA activity. Moreover, direct evidence from isotope tracer analysis confirmed less glucose entry into the TCA cycle. Together, these results demonstrate that HSulf-1 loss hindered the energy flow from glycolysis into TCA cycle.

Of note, it was originally hypothesized that these metabolic changes reflect a non-functional state of mitochondrial oxidative phosphorylation OXPHOS and that the high glycolytic rate in the tumors is due to impaired mitochondrial respiration [29]. Interestingly, our results showed that stable knockdown of HSulf-1 exhibited lower oxidative phosphorylation compared to NTC, while overexpression of HSulf-1 reversed this phenotype in TOV21G Cll cells. Because tumors arise through a Darwinian evolutionary process, the continuous absence of OXPHOS in tumor cells could dictate one or multiple reversible and/or irreversible adaptations that confer a growth advantage [30]. It is likely that the reduction of OXPHOS capacity of these tumor cell lines had been irreversibly diminished and could reflect on mitochondrial biogenesis and morphology as previously described [30]. Indeed, our results demonstrated that there is lower mitochondrial activity in HSulf-1-deficient cells which can be rescued by re-expression of HSulf-1. Interestingly, TEM analysis demonstrated a reduction in mitochondrial number and altered mitochondrial morphology (extended to round shape) in HSulf-1 deleted Sh1 cells. Alternatively, HSulf-1 overexpression in TOV21G cells resulted in a decrease in the mitochondrial activity and rescue of normal mitochondrial structure. These results demonstrated a fine interplay between glycolysis and mitochondrial metabolism regulated by HSulf-1. Collectively, our findings clearly established a strong connection between loss of HSulf-1 and reprogramming of metabolism, by inducing glycolysis, disrupting the energy flow to mitochondria and affecting mitochondrial morphology and activity.

These metabolic changes appear to be strongly associated with HSulf-1-HB-EGF mediated signaling resulting in c-Myc activation. Studies suggests that the metabolically active pathways are regulated by an upstream cascade of signaling mechanisms involving growth factor receptor tyrosine kinases (RTK) and this involvement of RTK is implicated in glucose homeostasis [31]. Previous reports from our lab and others unequivocally established the role of HSulf-1in modulating heparin-binding growth factor signaling leading to enhanced ERK activation [6]. Consistent with these observations our microarray analysis and *in vitro* results showed activation of EGFR-Ras-Raf-MAPK axis which culminates in ERK-mediated c-Myc activation. c-Myc has been established as a master regulator of metabolism and directly regulates the glycolytic pathway by activating Glut 1, PFK, PKM2, LDHA and enolase [32]. Additionally, c-Myc has been found to regulate mitochondrial function, biogenesis and morphology [33]. Indeed, our results showed that HB-EGF stimulation resulted in a time-dependent induction of c-Myc both in NTC and Sh1 cells. However, HB-EGF treatment upregulated the glycolytic enzymes more in the Sh1 cells compared to the NTC cells. Importantly, c-Myc inhibition markedly reduced the basal and HB-EGF-induced Glut 1, HK-II and LDHA levels in Sh1 cells. These results provide a plausible explanation of how loss of HSulf-1 mediated enhanced HB-EGF-growth factor signaling can activate c-Myc and as a consequence, directly alter glucose metabolism. However, we noticed that HB-EGF enhanced the cMyc target genes at early time points and tapered at 90 mins, while sustaining c-Myc expression. This possibly could be due to two reasons. It has been reported that growth factor stimulation results in biphasic effects leading to sudden increase in the target gene expression followed by a tapering but sustained effect [34, 35]. Second, it is possible that c-myc acts in conjunction with other transcriptional complex factor in regulating these genes. Thus availability of all the factors is essential for expression of Glut 1, HKII and LDHA. Limited availability of other factors and/or shorter half-life of the factors involved may be a reason for decreased expression of target genes at later time points.

Metabolic targeting for cancer therapy is currently under investigation and small molecules specifically inhibiting key glycolytic steps have been evaluated as an anti-cancer approach. However, therapeutic agents that target glycolysis do not always provide satisfying outcomes in clinical trials [36]. We report here that PG545 attenuated glucose uptake and lactate production through the inhibition of p-ERK, c-Myc and the key glycolytic enzymes in *vitro* and *in vivo*. Moreover, PG545 decreased tumor growth possibly by inducing apoptosis as evidenced by increased cleaved Caspase 3 and PARP. More intriguing is the possibility that PG545,that mimics the action of HSulf-1 by competing for growth factor binding and thus inhibiting HB-EGF mediated signaling has additional effects *in vivo* by targeting the tumor microenvironment [6]. It is noteworthy that PG545 treatment of the NTC cells expressing HSulf-1 had a more pronounced effect in downregulating the expression of the Glut1, HKII and LDHA, thus supporting the role of HSulf-1 as a modulator of glycolysis. Our study, therefore, has important implications for the development of ovarian cancer therapies targeting the altered metabolism, where tumor suppressor HSulf-1 is lost.

## MATERIALS AND METHODS

### Materials

PG545 supplied by Progen Pharmaceuticals Ltd (Brisbane, Australia). Antibodies used: HSulf-1, PFKFB3 (Abcam, MA, USA), Glut1 (Santa Cruz Biotechnology, CA, USA), HKII, ALDOC, PGAM, PKM2, LDHA, PDH, PDK1, Tubulin, GAPDH (GeneTex, CA, USA),c-Myc, p-ERK, T-ERK (Cell signal technology, MA, USA). Secondary antibodies anti-HRP-Rabbit, anti-HRP-Mouse antibodies were from BD Pharmingen (CA, USA). ATP and LDH assay kits were from Biovision (CA, USA) and Sigma-Aldrich (MO, USA).

### Cell lines and culture condition

H358 is a non-small cell lung carcinoma cell line derived from metastatic sites (http://www.pheculturecollections.org.uk/products/celllines/generalcell/detail.jsp?refId=95111733&collection=ecacc_gc), highly metastatic prostate cancer cell line PC3 [37]and HSulf-1 deficient,MDA231, a triple negative breast cancer cell line [15] were cultured in 10% RPMI-1640 and DMEM respectively. HSulf-1^+/+^ and HSulf-1^−/−^ cells were isolated from HSulf-1^+/+^ and HSulf-1^−/−^ mice and immortalized as previously described [38]. MEFs were maintained in DMEM supplemented with 10% FBS and 650 μg/ml of G418. H358, PC3 and MDA231 are HSulf-1 deficient cells.

### Transfection, plasmids and shRNA

Transient Transfection: pcDNA-HSulf-1 plasmid was transiently overexpressed in H358, PC3 and MDA231 cells using Lipofectamine Plus (Invitrogen, Grand Island, NY) according to the manufacturer's instruction.

#### Generation of HSulf-1 overexpression stable clones

Upon 60% confluency, HSulf-1 deficient ovarian clear cell cancer line TOV21G cells [10] were transfected with pcDNA3.1-WT-HSulf-1 using lipofectamine-LTX-plus. After 24hr of transfection, cells were selected on 100μg/ml G418 and individual clones were picked using propagated as HSulf-1 overexpressing TOV21G stable clones.

#### Generation of HSulf-1 downregulated Stable clone

OV202 stable clones OV202Sh1 and OV202Sh2 were generated as previously reported [16].

#### Rescue of HSulf-1 in Sh1 cells

As HSulf-1ShRNA targets the 3′UTR of HSulf-1 [16], HSulf-1 expression in Sh1 cells was rescued with CMV-driven WT-HSulf-1construct in OV202Sh1cells We used OV202Sh1-clone-7 as our rescue model and designated as Cl7 throughout the study. OV202 clones were cultured in minimal essential medium supplemented with 20% FBS with proper antibiotic selection.

### Glucose uptake

Glucose uptake of the live cells was measured using 2-NBDG (Cayman Chemicals, Michigan, USA) according to manufacturer instruction. Fluorescent intensities were calculated using Image J software.

### Immunoblot analysis

Immunoblot analysis was carried out as previously described [39].

### ATP, LDH assay

Cellular ATP content and LDH activity were measured using kits from BioVision and calculated as per manufacturer's instruction.

### Quantitative real time PCR

Quantitative real-time PCR was carried out with SYBR-Green PCR Master Mix (Applied Biosystems, NY, USA), using specific primers with GAPDH or 18S ribosomal subunit as an internal control as previously described [21].

### Mitotracker staining and confocal microscopy

Cells (5×10^4^/500μl) were seeded in a 4-well chamber slide, incubated overnight and stained with 100nM mitotracker red for 30 minutes. Cells were then washed, fixed and mounted with DAPI and analyzed under Zeiss LSM 510 confocal microscope. Intensity of red florescence was measured using Image J software.

### Transmission electron microscopy

Cultured cells were washed and fixed in Trumps fixative containing 4% formaldehyde and 1% glutaraldehyde in a phosphate buffer pH ~7.3, post-fixed in 1.0% OsO4, dehydrated with ethanol gradation, and transitioned into propylene oxide for infiltration and embedding into spurr epoxy resin. Ultrathin sections were cut onto grids, stained with uranyl acetate and lead citrate, and examined with JEM-1400 (JEOL USA) transmission electron microscope and digitally photographed.

### Microarray expression data analysis

OV202NTC, Sh1 and Sh2 cells in triplicates were profiled using Illumina Whole Genome DASL assay as previously described [21].

### Liquid chromatography/Mass Spectrometry (LC/MS, LC/MS2) and Gas chromatography/Mass Spectrometry (GC/MS)

The samples destined for LC/MS, LC/MS2 and GC/MS analysis were prepared and analyzed as previously described [21].

### Metabolic tracing using GC-MS

#### Metabolic extraction

At ~80% confluence, OV202 cells (NTC, Sh1, Sh2) were cultured with U-^13^C_6_ glucose, then extracted, derivatized and analyzed by GC/MS as previously described [40, 41].

### Animal study

Both the animal experiments were performed with the approved protocol of Mayo Clinic Institutional Animal Care and Use Committee (IACUC).

#### FDG-PET imaging

Female athymic mice (5-6 weeks) were injected intraperitoneally (i.p.) with 5×10^6^ OV202 NTC (*n* = 5) and OV202 Sh1 (*n* = 5) cells. After 2 weeks, mice were anesthetized by isoflurane and 500 μCi of ^18^FDG was administered intravenously (i.v.) via the tail vein. After 45 minutes of ^18^FDG injection, micro-PET imaging was acquired using Siemens Inveon PET/CT scanner (Siemens Medical Solutions, Molecular Imaging, TN, USA). Regions of interest in the tumor were quantified using the PMOD Biomedical Image Quantification and Kinetic Modeling Software (PMOD Technologies, Switzerland).

#### Tumor xenograft

Female athymic mice (5-6 weeks) were randomized in two groups (*n* = 10) and OV202 Sh1 cells (4×10^6^) were injected i.p. PG545 treatment commenced 5 days after tumor inoculation at 20mg/kg, i.p., twice a week. Treatment continued for the next 4 weeks. However control mice were sacrificed during week 3 as the tumor burden exceeded 10% of their body weight. Mice were sacrificed according to standard IACUC procedure and the tumors and tissues were excised and preserved either in formalin or −80°C.

#### Statistical analysis

All the results were expressed as the mean ± S.D. data obtained from three separate experiments. All statistical analysis was evaluated using Graph pad Prism software (San Diego). Data were analyzed by the paired t test, and P values less than 0.05 or mentioned otherwise, was considered statistically significant.

## SUPPLEMENTARY MATERIAL FIGURES




