# Supplementary material for: HSulf-1 deficiency dictates a metabolic reprograming of glycolysis and TCA cycle in ovarian cancer
Source: Oncotarget. 2015 Sep 10;6(32):33705–19. doi: 10.18632/oncotarget.5605 (PMC4741796; doi:10.18632/oncotarget.5605)
Supplement: Supplementary file 1 [file oncotarget-06-33705-s001.pdf]

HSulf-1 deficiency dictates a metabolic reprograming of glycolysis and TCA cycle in ovarian cancer

Supplementary Material

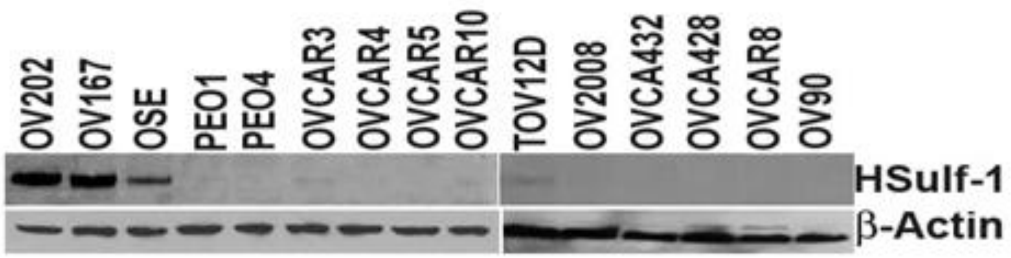

Fig S1: Immunoblot analysis of HSulf-1 expression in ovarian cancer cell lines. GAPDH is shown as loading control

S2A and B are videos

Fig S2: Enhanced glucose uptake using ^{18}FDG in OV202NTC and Sh1 xenograft. Two weeks old tumor xenograft (n=5) were injected with ^{18}FDG and 45 min later scanned for the uptake using Siemens Inveon PET/CT scanner.

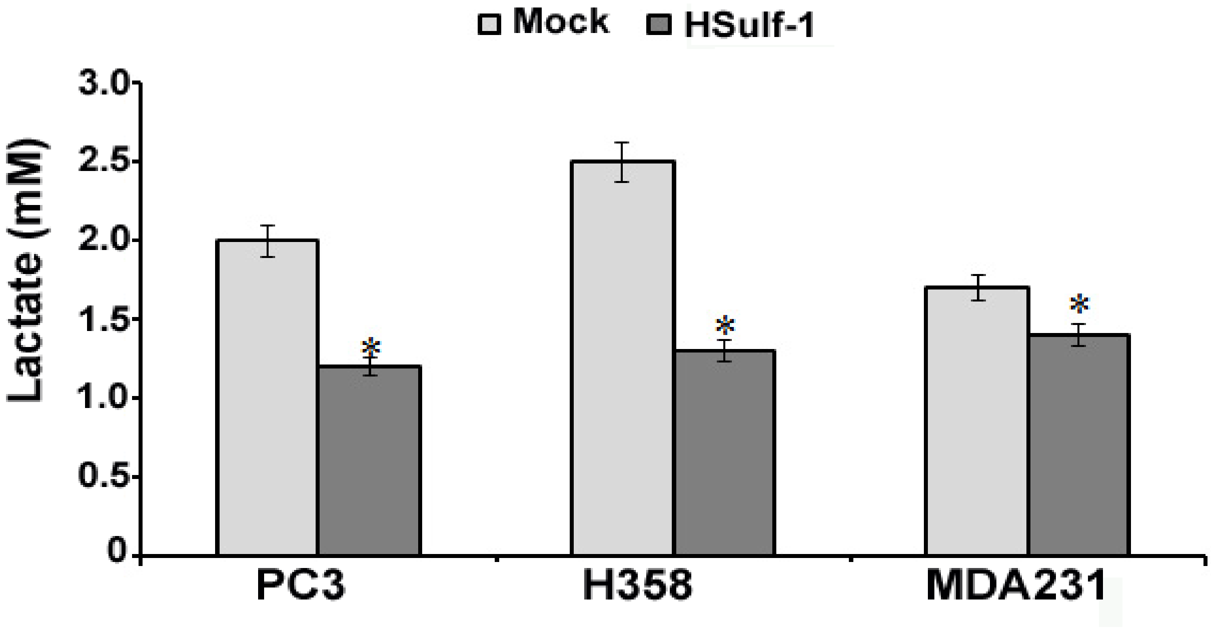

Fig S3: HSulf-1 overexpression reduced lactate secretion in cancer. Lactate secretion after 48 hr of transient overexpression of HSulf-1 in PC3, H358 and MDA231 cells, where *p<0.05.

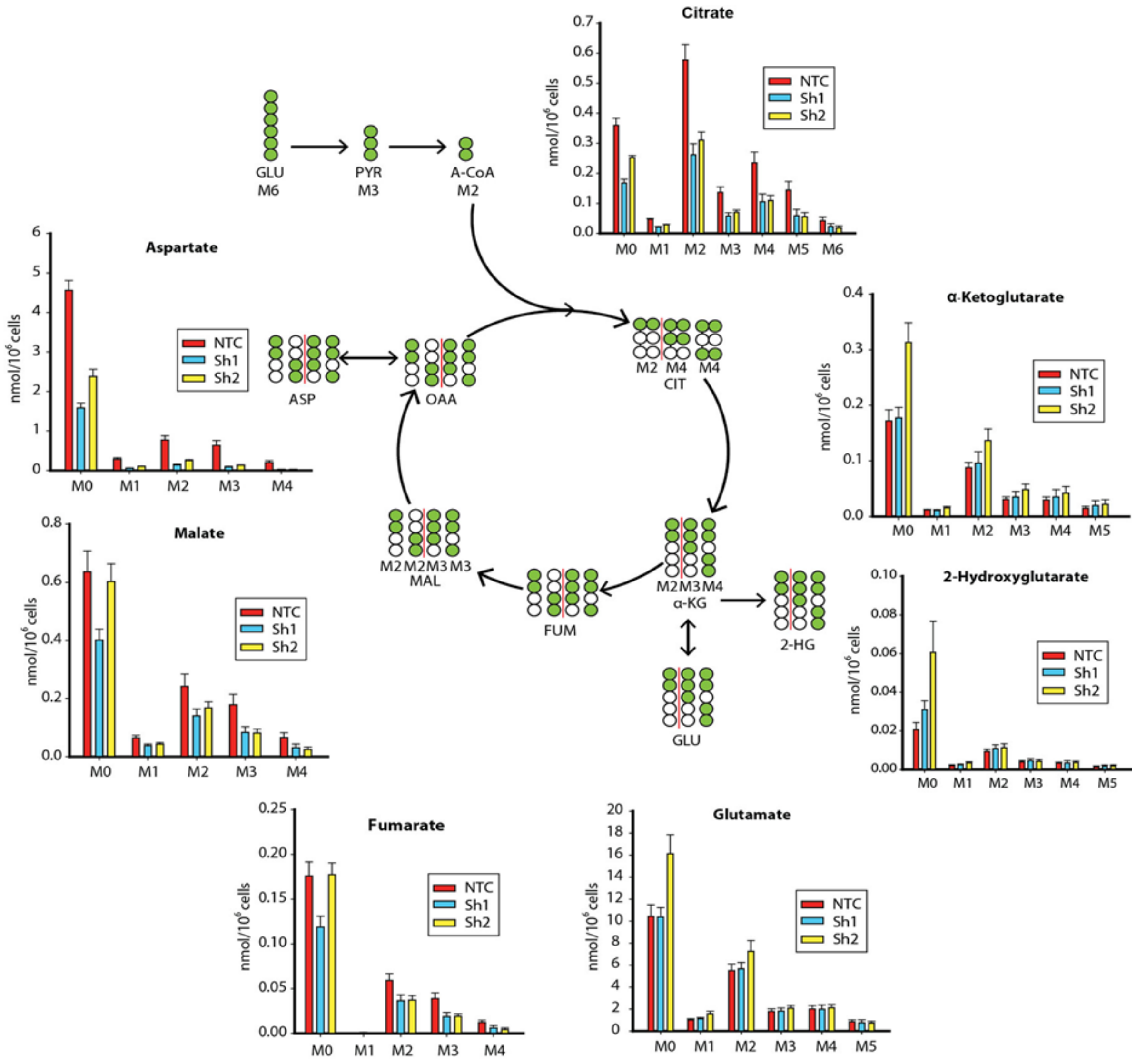

-

Fig S4: Glucose contribution to TCA cycle fluxes in ovarian cancer cells using 13-C GC-MS based isotope tracer analysis using labeled U-$^{13}C_6$ glucose. Details of the flux are described in the main text.

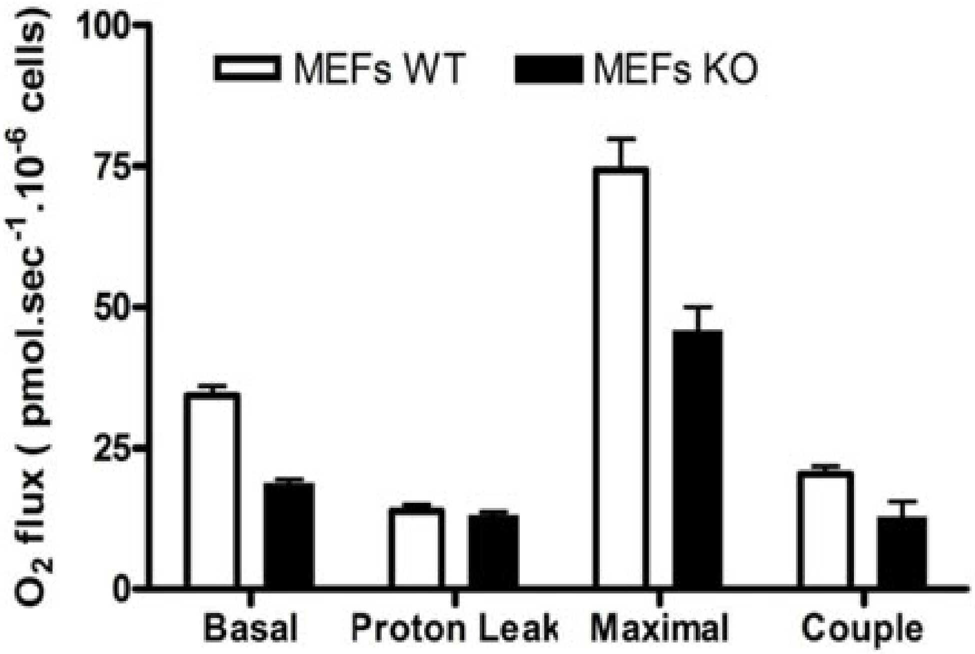

Fig.S5: Enhanced oxygen consumption in HSulf-1 deficient cells. Oxygen consumption rates were measured using respirometry (Oroboros Oxygraph-O2K) and Data Lab Software (Oroboros). Intact equal MEK WT and KO cells (2 x 10^6) were added in oxygraph and the basal respiration rate was measured. Subsequently, oligomycin, FCCP and rotenone were added and leak, maximal and residual oxygen consumption were measured.
